# Supplementary material for: Replicable generation and stable maintenance of rhesus macaque iPSCs for in vitro modeling of genetic frontotemporal dementia
Source: Front Cell Dev Biol. 2026 Jul 30;14:1833030. doi: 10.3389/fcell.2026.1833030 (PMC13469415; doi:10.3389/fcell.2026.1833030)
Supplement: Supplementary file 1 [file DataSheet1.pdf]

Supplementary Table 1. Media formulations.

Medium	Reagent	Concentration	Vendor	Cat. No.
Biopsy	DMEM/F12	99%	Thermo Fisher Scientific	SH30023
	Pen-Strep	1%	Thermo Fisher Scientific	15140122
Digestion	hi-glucose DMEM	80%	Thermo Fisher Scientific	11960044
	FBS	20%	Peak Serum	PSFB4
	Collagenase type I	0.4 mg/mL	Worthington Biochemical	LS004194
	DNAse I	2 mg/mL	Sigma-Aldrich	DN25
	Pen-Strep	1%	Thermo Fisher Scientific	15140122
Fibroblast	DMEM/F12	80%	Thermo Fisher Scientific	SH30023
	FBS	20%	Peak Serum	PSFB4
	Sodium Pyruvate	1%	Lonza Bioscience	BEBP13-115E
	Non-essential amino acids	1%	Gibco	11140050
	FGF-2	20 ng/mL	Bio-Techne	BT-FGFB-100
	Pen-Strep	1%	Thermo Fisher Scientific	15140122
N2B27	DMEM/F12	50%	Thermo Fisher Scientific	SH30023
	Neurobasal	50%	Thermo Fisher Scientific	21103049
	B27 Supplement	1X	Thermo Fisher Scientific	12587010
	N2 Supplement	1X	Thermo Fisher Scientific	17502048
	GlutaMAX	2 mM	Thermo Fisher Scientific	35050061
	Non-essential amino acids	0.1 mM	Gibco	11140050
	BME	0.1 mM	Thermo Fisher Scientific	21985023
	BSA	2 mg/mL	Sigma-Aldrich	A8806
	FGF-2	20 ng/mL	Bio-Techne	BT-FGFB-100
	IWR1	2.5 µM	STEMCELL Technologies	72562
	CHIR99021	3 µM	Tocris	4423
UPPS	StemMACS iPS Brew XF	base	Miltenyi Biotec	130-104-368
	endo-IWR1	1 µM	STEMCELL Technologies	72562
	CHIR99021	0.5 µM	Tocris	4423
E11+Bu	DF3S	base	Thermo Fisher Scientific	custom order
	Transferrin	10.7 µg/mL	Thermo Fisher Scientific	2914-HT-001G
	Insulin	20 µg/mL	Sigma-Aldrich	I9287-5ML
	FGF-2	100 ng/mL	Bio-Techne	BT-FGFB-100
	GlutaMAX	1X	Thermo Fisher Scientific	35050061
	Chemically Defined Lipids	1X	Thermo Fisher Scientific	11905031
	rhNodal	50 ng/mL	R&D Systems	3218-ND
	Glutathione	1.94 µg/mL	Sigma-Aldrich	G4251
	Sodium Butyrate	100 µM	STEMCELL Technologies	72242
E12+IWR1	DF3S	base	Thermo Fisher Scientific	custom order
	Transferrin	10.7 µg/mL	Thermo Fisher Scientific	2914-HT-001G
	Insulin	20 µg/mL	Sigma-Aldrich	I9287-5ML
	FGF-2	100 ng/mL	Bio-Techne	BT-FGFB-100
	GlutaMAX	1X	Thermo Fisher Scientific	35050061
	Chemically Defined Lipids	1X	Thermo Fisher Scientific	11905031
	rhNodal	50 ng/mL	R&D Systems	3218-ND
	Glutathione	1.94 µg/mL	Sigma-Aldrich	G4251
	TGFβ	1.7 ng/mL	R&D Systems	240-B
	IWR1	2.5 µM	STEMCELL Technologies	72562
Complete	DMEM/F12	90%	Thermo Fisher Scientific	SH30023
	FBS	10%	Peak Serum	PSFB4
Neural Induction	STEMdiff Neural Induction Medium	base	STEMCELL Technologies	08581
	STEMdiff SMADi Neural Induction Supplement	0.5 mL	STEMCELL Technologies	08581
Neural Freezing	DMSO	20%	Sigma-Aldrich	D8418
	KnockOut Serum Replacement	80%	Thermo Fisher Scientific	10828010

Supplementary Table 2. Electroporation parameters and outcomes. The percentage of live cells (%Live) and eGFP-expressing cells (%eGFP) post-electroporation are shown for each experimental replicate (rep 1, rep 2), fibroblast line (W4, M4, W6, M6), and Neon transfection program. Data were visually inspected and cutoffs were rendered to reflect programs with the highest %eGFP and relatively high %Live. The Neon programs with both criteria above the cutoff were identified (highlighted in yellow). Asterisks (*) represent replicates in which the %eGFP was above the cutoff. *Abbreviations: eGFP, enhanced green fluorescent protein; no., number of pulses; rep, replicate; v, voltage.*

	**Program Conditions**	**W4**				**M4**				**W6**				**M6**			
**Program #**	**v, width, no.**	**%Live, rep 1**	**%eGFP, rep 1**	**%Live, rep 2**	**%eGFP, rep 2**	**%Live, rep 1**	**%eGFP, rep 1**	**%Live, rep 2**	**%eGFP, rep 2**	**%Live, rep 1**	**%eGFP, rep 1**	**%Live, rep 2**	**%eGFP, rep 2**	**%Live, rep 1**	**%eGFP, rep 1**	**%Live, rep 2**	**%eGFP, rep 2**
1	none	97.4	0.5	91.5	0.49	97.9	0.51	87.9	0.6	99.6	0.48	99.7	0.50	96.2	0.52	98.9	0.51
2	1400, 20, 1	71.9	0.8	86.70	22.70	90.3	3.25	73.9	1.04	99.7	7.32	92.7	8.74	93.1	18	98.4	4.67
3	1500, 20, 1	52	2.4	77.90	22.90	88.3	3.72	75.4	3.53	98.7	15.6	90.5	13.60	90.7	26.6	97.8	12.5
4	1600, 20, 1	49.4	9.45	88.40	0.65	83.8	4.78	61.8	2.13	98.1	17.7	89.1	10.90	83.8	39.2	97.3	10
5	1700, 20, 1	51.6	14.4*	85.90	1.79	75.7	6.06*	75.1	9.84*	93.7	34.4	84.4	25.50	75.4	52.9*	94	20.4*
6	1100, 30, 1	52.8	0.89	80.3	23.10	93.9	1.97	77.6	1.14	99.7	4.35	92.3	4.84	96	10.3	96.8	1.46
7	1200, 30, 1	81.5	2.44	83.9	4.17	92.3	2.67	75.1	1.79	99.5	7.23	92.6	10.90	92.4	24	95.1	3.84
8	1300, 30, 1	68.8	1.44	76.00	22.00	88.5	3.68	68.2	3.86	99.2	10.3	90.3	19.40	87	32.2	96	3.54
9	1400, 30, 1	59.5	9.09	74.10	27.90	79.2	4.89	59.9	0.12	95.3	28.6	91.8	21.80	72.9	48.8	87.5	20.2*
10	1000, 40, 1	75.7	1.53	80.10	21.40	94	1.85	75.7	0.9	99.6	6.03	90.0	4.87	94.8	11.4	95.4	2.18
11	1100, 40, 1	64.6	2.35	75.70	27.90	91.1	2.44	75.4	0.48	99	12	99.4	3.78	91	22.7	95.7	6.37
12	1200, 40, 1	54.1	2.54	61.40	28.90	87.3	3.48	78.8	6.39	97.9	17.5	89.9	27.00	79.7	39.3	94.5	2.82
13	1100, 20, 2	72.9	2.56	85.30	5.00	92.1	2.29	79.8	2.84	98.8	15.6	93.4	9.60	91.9	20.2	95.9	4.22
14	1200, 20, 2	45	4.61	74.4	31.2*	85.9	3.73	76.5	4.98	96.2	24.7	91.1	20.90	84.2	33.9	94.6	9.24
15	1300, 20, 2	69	8.92	66.9	27.5	74.1	5.67*	77.8	11.4*	86.5	48.6*	83.3	35.40*	70	49.3	93.7	19.1
16	1400, 20, 2	52.6	10.8*	58.8	33.7*	60.5	7.34*	79.6	0.42	70.7	63.1*	87.5	30.60	87.5	58.3*	87.9	21.9*
17	850, 30, 2	83.9	0.49	81.2	14.4	94.9	1.64	76.6	0.48	99.8	4.23	91.4	2.35	96.3	6.63	96	1.01
18	950, 30, 2	75.6	1.25	77.80	30.5*	97.8	0.44	76.3	1.23	97.6	19.4	89.1	7.24	93.1	17.2	95.4	2.75
19	1050, 30, 2	75.1	4.92	83.20	2.00	88.1	2.58	78	0.06	95.8	23	89.1	20.80	84.2	32.4	94.5	9.49
20	1150, 30, 2	46.2	6.73	34.1	34.9*	73	5.14	71.9	0.6	83.2	47.5	81.8	40.30*	88.5	50.7	96.8	0.75
21	1300, 10, 3	59.6	0.62	77.8	20.3	92.9	2.14	70	3.46	98.8	14.5	90.7	4.79	94.2	13.4	95	5.86
22	1400, 10, 3	63.9	2.9	66.9	29.1	88.2	3.09	76.4	5.64	95.1	30.2	89.8	18.70	86.3	28.8	95.5	0.9
23	1500, 10, 3	44.6	0	73.2	11.2	82.9	4.3	72.9	9.49*	92.5	36	83.0	31.20	81.1	39.9	94.2	14.5
24	1600, 10, 3	53.4	19.1*	57.9	29.4	74.1	4.91	68.4	0.81	84.5	51.2*	78.7	47.80*	77.6	50.9*	97	0.68
Cutoff		>50%	>10%	>55%	>30%	>60%	>5.5%	>70%	>9%	>70%	>48%	>78%	>35%	>75%	>50.9%	>85%	>20%

Supplementary Table 3. List of plasmids used for oriP/EBNA1 reprogramming trials with vendor info, promoter(s) for transgene expression, and transgene(s) contained within the plasmid.

**Plasmid**		**pEP4 E02S ET2K**	**pEP4 E02S EN2K**	**pCEP4-M2L**	**pEP4 E02S EM2K**	**pEP4 E02S EN2L**	**pSimplemiR 302/367**	**pSP6-EBNA (2A+DBD)**
**Addgene Plasmid #**		20927	20925	20926	20923	20922	98748	98749
**Promoter(s)**		EF1α	EF1α	CMV (*c-MYC*)<br>IRES (*LIN28A*)	EF1α	EF1α	EF1α	SP6
**Transgene(s)**		*POU5F1*<br>*SOX2*<br>*KLF4*<br>*SV40LT*	*POU5F1*<br>*SOX2*<br>*KLF4*<br>*NANOG*	*c-MYC*<br>*LIN28A*	*POU5F1*<br>*SOX2*<br>*c-MYC*<br>*KLF4*	*POU5F1*<br>*SOX2*<br>*NANOG*<br>*LIN28A*	*MIR302A*<br>*MIR367*	*EBNA1*
**Trial #**	**1**	x	x	x			x	x
	**2**	x	x	x			x	x
	**3**	x	x	x			x	x
	**4**	x	x	x			x	x
	**5**	x	x	x			x	x
	**6**	x	x	x			x	x
	**7**	x			x	x	x	x
	**8**	x			x	x	x	x
	**9**	x			x	x	x	x
	**10**	x			x	x	x	x
	**11**	x			x	x	x	x
	**12**	x			x	x	x	x
	**13**	x			x	x	x	x

Supplementary Table 4. Passage numbers of RhiPSC lines at time of characterization, cloning efficiency experimentation, or NPC differentiation. *Abbreviations: AP, alkaline phosphatase; ICC, immunocytochemistry; N/A, not applicable; NPC, neural progenitor cell; NR, not recorded; PCR, polymerase chain reaction; RhiPSC, rhesus macaque induced pluripotent stem cell; RT, reverse transcription; STR, short tandem repeat.*

RhiPSC Line	AP	ICC	RT-PCR	Flow	Episomal Plasmid PCR	*MAPT* Genotyping	STR	Mycoplasma	Karyotype	Teratoma	Cloning Efficiency	NPC Differentiation
W4 Clone 14.06	P14	P15	P9	P25	P14	NR	P16	P15	P10	P16	P20	P17
W4 Clone 16.10	P14	P15	P9	P18	P14	NR	P16	P15	P10	P16	N/A	N/A
W6 Clone 5.07	P22	P23	P13	P9	P16	P16	P16	P20	P12	P15	P19	P15
W6 Clone 15.03	P9	P9	P14	P9	P18	P18	P19	P7	P13	P16	N/A	P16
M4 Clone 14.09	P10	P11	P14	P20	P14	P14	P17	P11	P18	P21	P20	P17
M4 Clone 16.01	P10	P11	P14	P10	P14	P14	P17	P11	P18	P21	N/A	N/A
M6 Clone 16.02	P16	P17	P16	P16	P18	P19	P22	P22	P15	P22	P23	P21
M6 Clone 4.04	P14	P15	P14	P14	P14	P17	P18	P19	P13	P22	N/A	N/A

Supplementary Table 5. Primer sequences.

Amplicon	Forward (5’-3’)	Reverse (5’-3’)	Amplicon Size (base pairs)
ACTB	CTA CCA TGA GCT GCG TGT GG	GTA CAT GGC TGG GGT GTT GA	130
EBNA1	ATC GTC AAA GCT GCA CAC AG	CCC AGG AGT CCC AGT AGT CA	666
KLF4	GTC CCG GGG ATT TAT AGC TCG	TGG TAA GGT TTC TCA CCT GTG T	276
LIN28A	ATC AAA AGG AGA CAG GTG CTA C	TCC CGA AAG TAG GTT GGC TT	180
MAPT	GGG CAC AGG GAT TAC GAA TTG	CAC CCC CAG TCT CTT GAA ATC	1052
MYC	GGT AGT GGA AAA CCA GCC TCC	GCA GTA GAA ATA CGG CTG CAC	103
NANOG	GGA TCC AGC TTG TCC CCA AA	AGG AAG GAA GAG GAG AGA CAG T	161
oriP	TTC CAC GAG GGT AGT GAA CC	TCG GGG GTG TTA GAG ACA AC	544
POU5F1	GCC CGA AAG AGA AAG CGA AC	CAC ACT CGG ACC ACA TCC TTC	146
SOX2	GGT AGG AGC TTT GCA GGA AGT	CCA ACG ATG TCA ACC TGC ATG	428

Supplementary Table 6. Antibody information.

	Primary Antibody	Clonality	Host Species	Dilution	Vendor	Cat. No.	Secondary Antibody	Dilution	Vendor	Cat. No.
ICC	OCT3/4	monoclonal	mouse	1:1000	Santa Cruz	sc-5279	Alexa fluor donkey anti-mouse 594	1:1000	Invitrogen	A-10037
	SOX2	monoclonal	goat	1:1000	Novus Biologicals	AF2018	Alexa fluor donkey anti-goat 488	1:1000	Invitrogen	A-11055
	NANOG	monoclonal	rabbit	1:1000	Cell Signaling Technologies	4903	Alexa fluor donkey anti-rabbit 647	1:1000	Invitrogen	A-31573
	PAX6	polyclonal	rabbit	1:500	Biolegend	PRB-278P	Alexa fluor donkey anti-rabbit 594	1:500	Invitrogen	A-21207
	NESTIN	monoclonal	mouse	1:500	Thermo Fisher Scientific	MA1-110	Alexa fluor donkey anti-mouse 488	1:500	Invitrogen	A-21202
Flow Cytometry	OCT3/4	monoclonal	mouse	1:100	Santa Cruz	sc-5279	APC goat anti-mouse IgG	1:500	Biolegend	405308
	SOX2	monoclonal	rabbit	1:100	Cell Signaling Technologies	3579	goat anti-rabbit IgG (H+L) cross absorbed, APC	1:500	Thermo Fisher Scientific	A-10931
	NANOG	monoclonal	rabbit	1:100	Cell Signaling Technologies	4903	goat anti-rabbit IgG (H+L) cross absorbed, APC	1:500	Thermo Fisher Scientific	A-10931

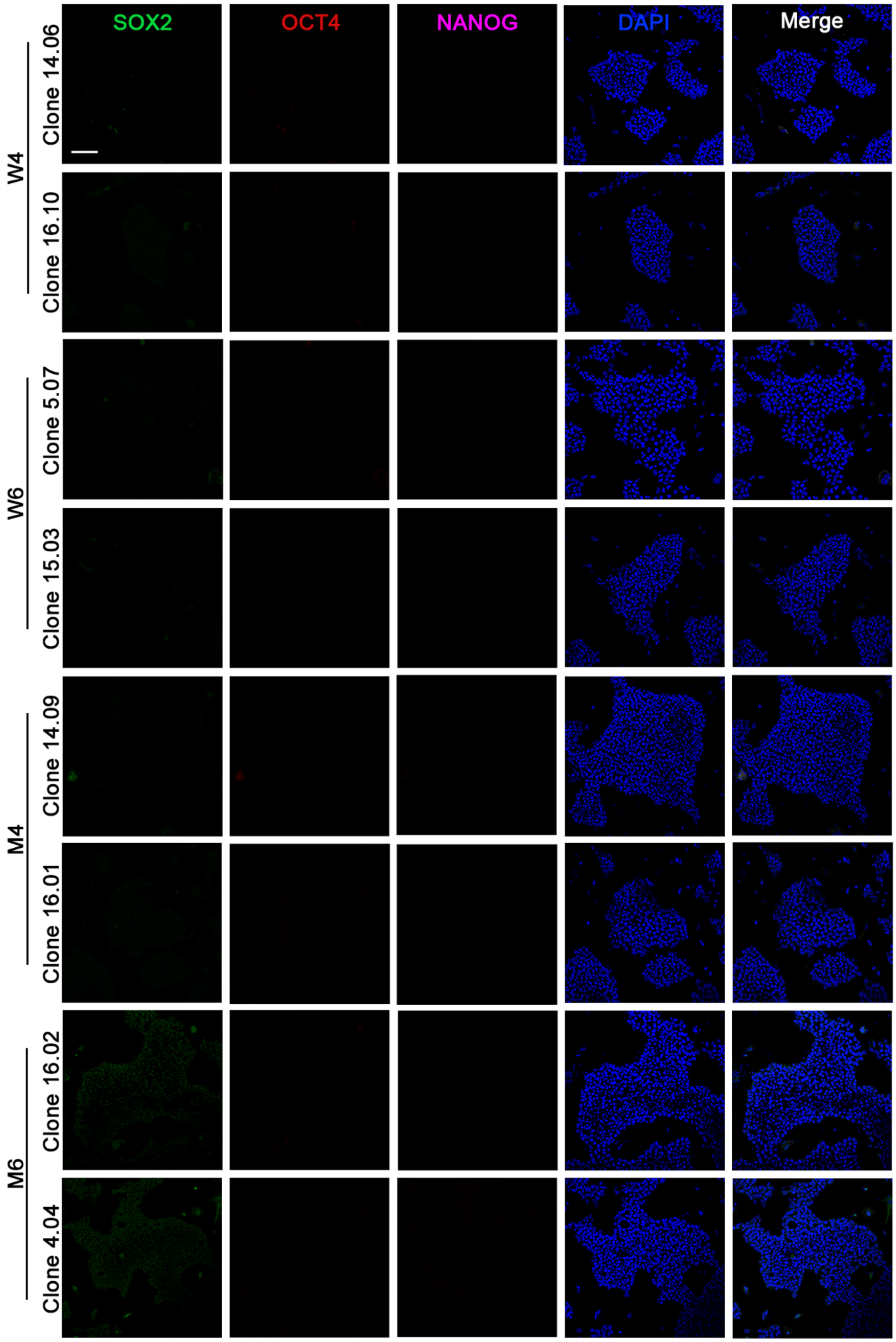

Supplementary Figure 1. Primary antibody-omitted negative control images for SOX2 (green), OCT4 (red), and NANOG (far red) immunostaining in all eight RhiPSC clones. Cells were counterstained with DAPI (blue). Scale bar, 100 µm.

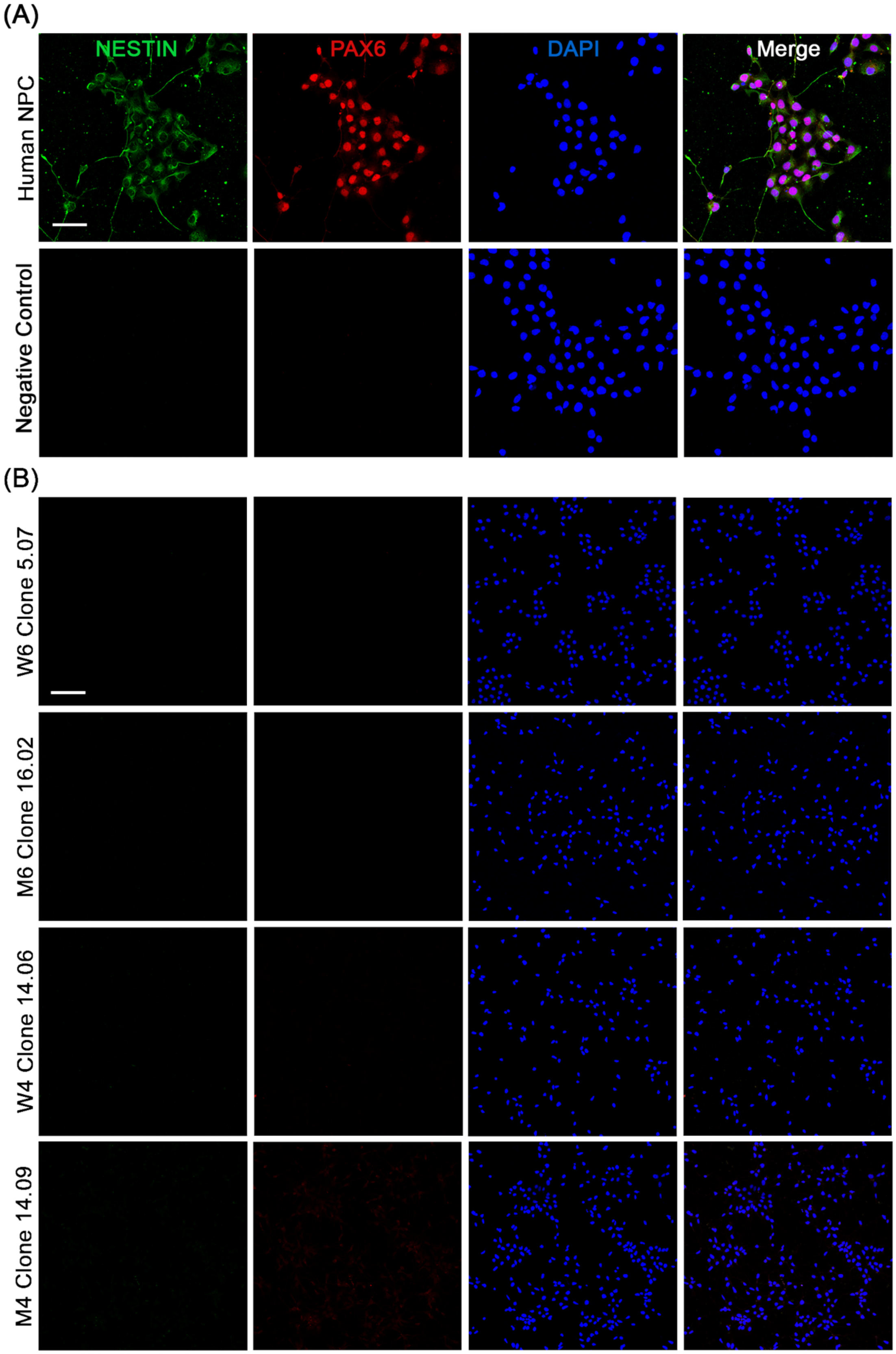

Supplementary Figure 2. (A) A human iPSC-derived neural progenitor cell (NPC) line carrying *MAPT* R406W was used as a positive control for NESTIN (green) and PAX6 (red) immunostaining. Below are the corresponding primary antibody-omitted negative control immunofluorescence images from this human NPC line. (B) Primary antibody-omitted negative control images for NESTIN and PAX6 in four RhNPC clones. All cells were counterstained with DAPI (blue). Scale bars, 100 µm.

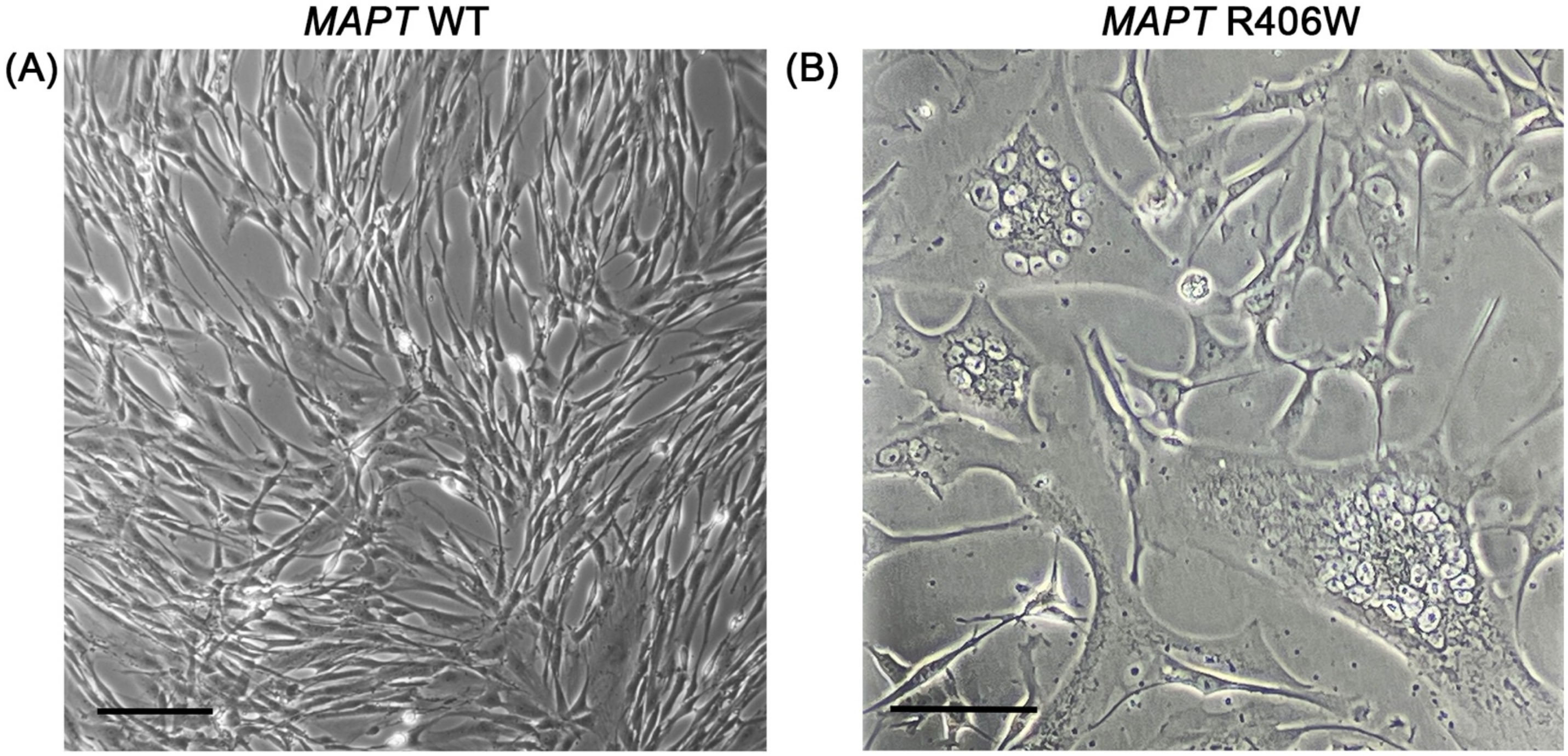

Supplementary Figure 3. Representative images of (A) *MAPT* wild type (WT) fibroblasts at P5 from W4 at P5 and (B) *MAPT* R406W fibroblasts at P3 from M2. Scale bars, 100 µm.

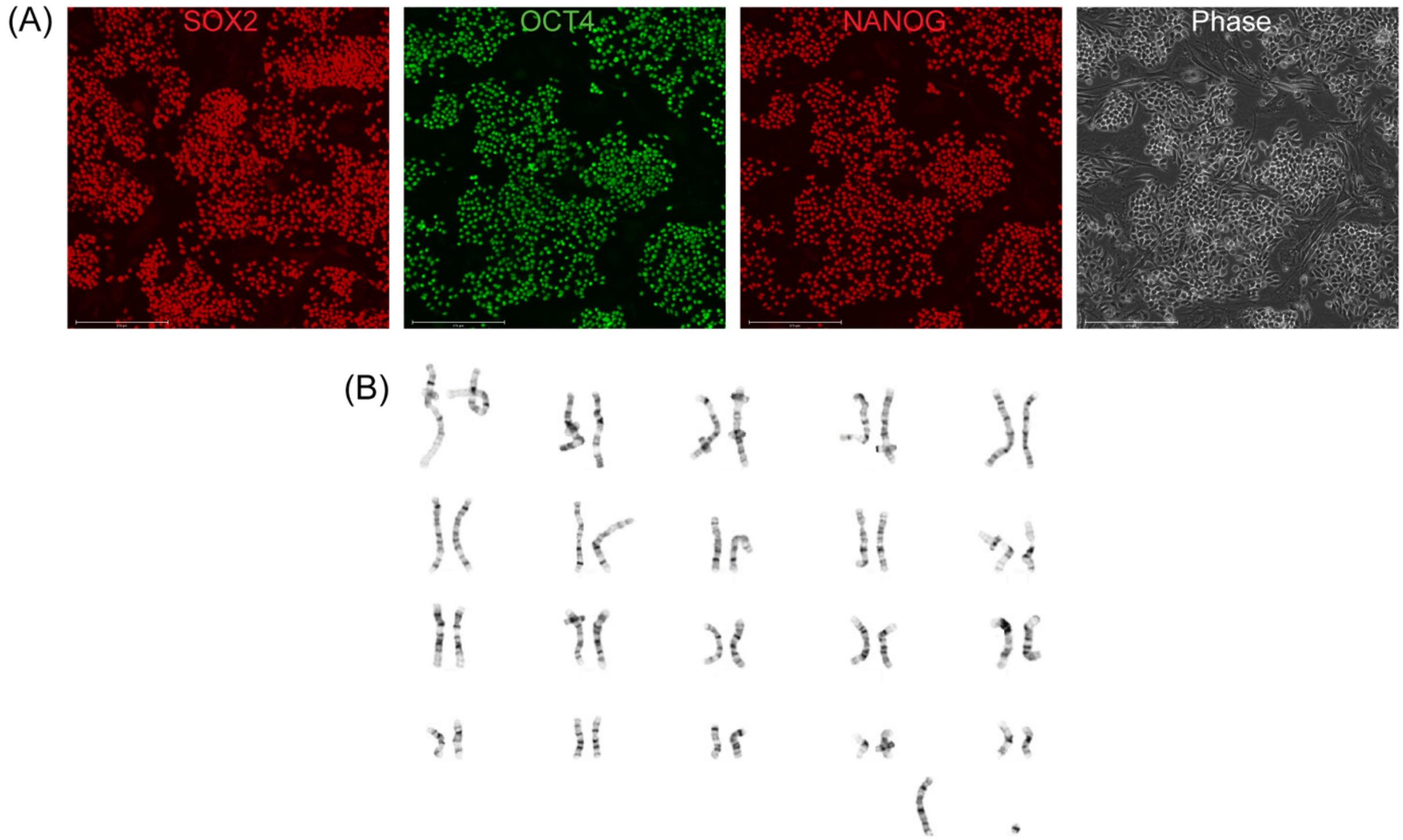

Supplementary Figure 4. RhiPSCs derived from animal M4 in episomal reprogramming trial 1 and maintained in E12+IWR1 medium. (A) RhiPSCs expressed pluripotency proteins SOX2, OCT4, and NANOG, yet cells within colonies were not tightly compacted. (B) Abnormal karyotype: 42,XY,der(6)t(3;6)(q;q). Scale bars, 275 μm.

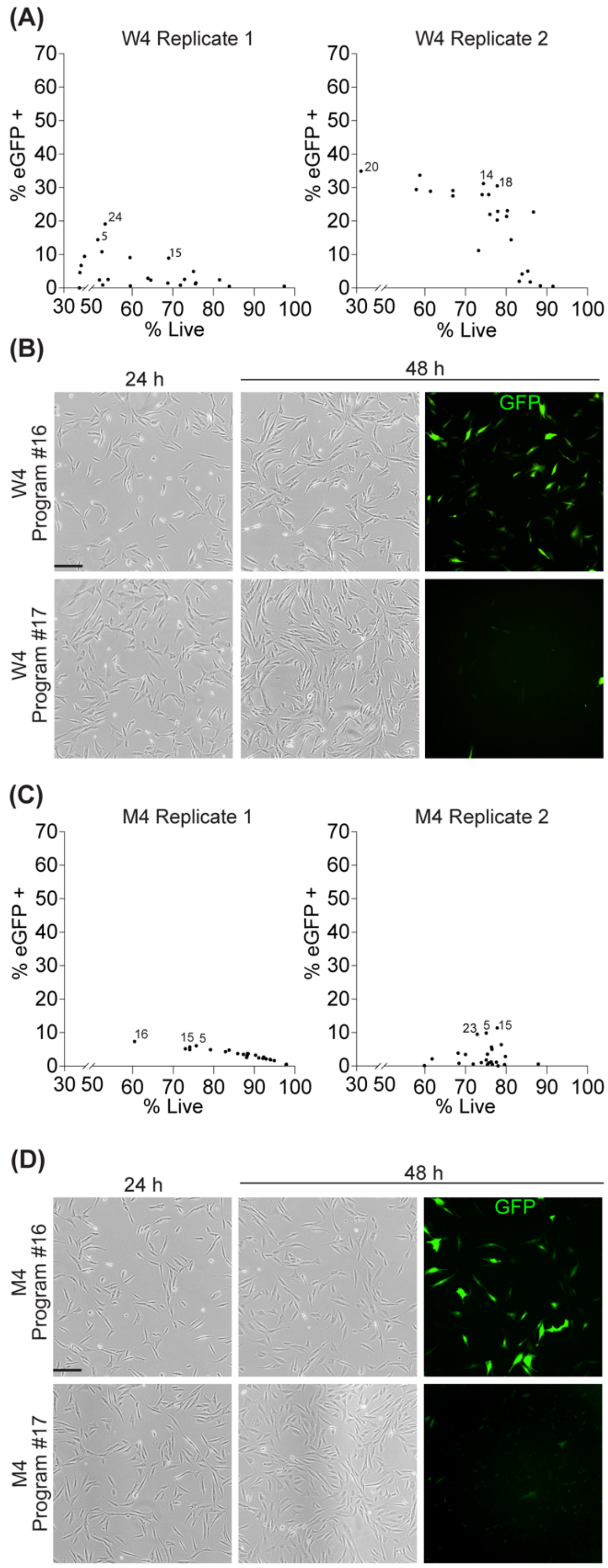

Supplementary Figure 5. Optimization of electroporation efficiency for fibroblast lines W4 and M4 using the Neon Transfection system. The percentage of eGFP-expressing cells (% eGFP+; y-axes) were plotted against the percentage of live cells (% live; x-axes); each dot represents data from each of the 24 different Neon programs. (B, D) Representative phase contrast images of fibroblasts lines W4 (B) and M4 (D) demonstrating post-electroporation cell survival at 24 and 48 h on Neon program #16 (an optimal program) and program #17 (a suboptimal program). Corresponding immunofluorescence images were taken 48 h post-electroporation to demonstrate the eGFP+ population resulting from Neon programs #16 and #17. Scale bars, 100 µm.

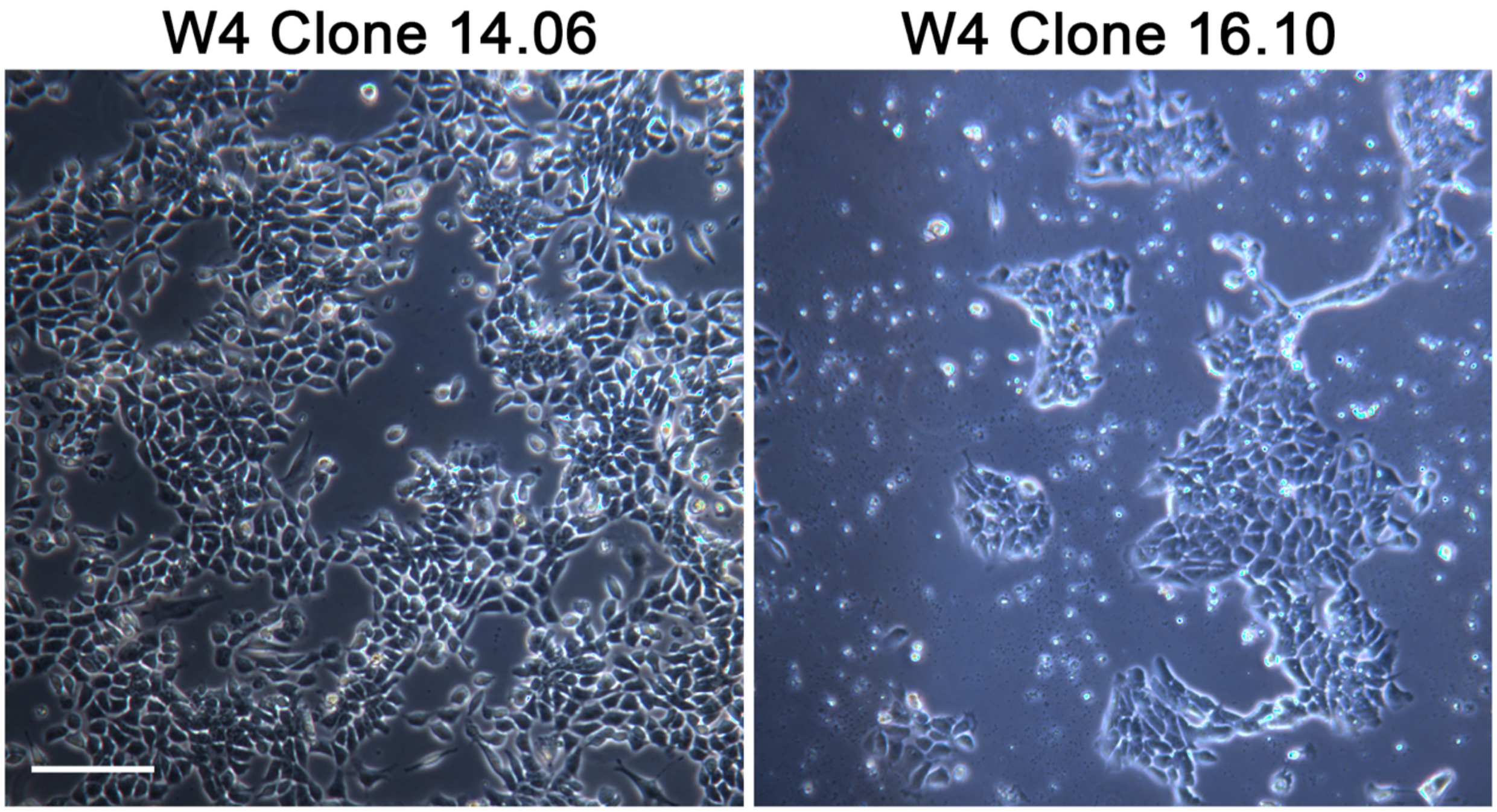

Supplementary Figure 6. Representative images of W4 RhiPSCs Clone 14.06 at passage 15 and W4 Clone 16.10 at passage 16 on laminin-521. Scale bar, 100 µm.

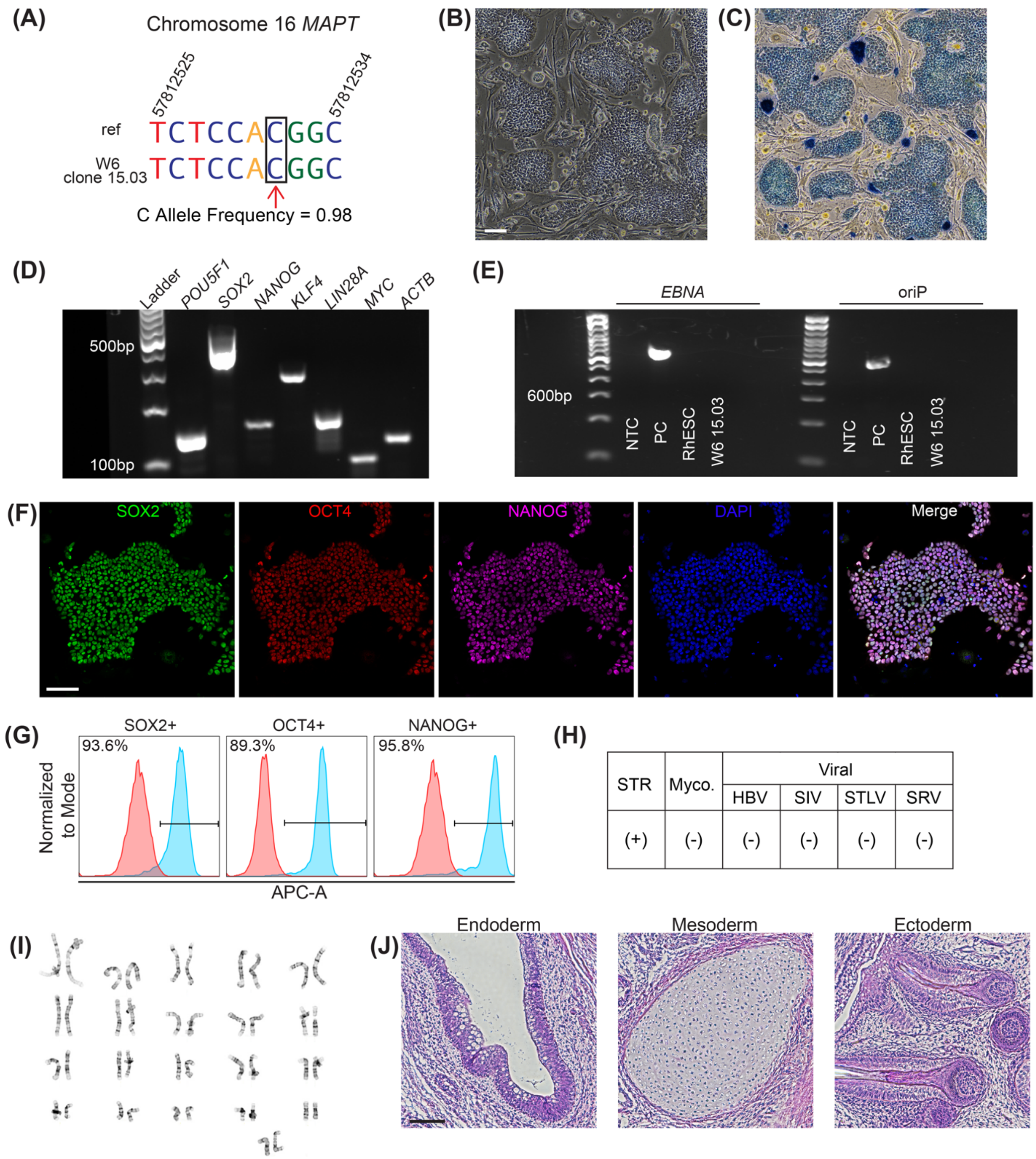

Supplementary Figure 7. Characterization of RhiPSC line W6 clone 15.03. (A) Amplicon sequencing of *MAPT* R406W with wild-type C allele indicated in the black box with a red arrow compared to reference genome (ref). (B) Phase contrast image of colonies grown on MEFs and (C) positive alkaline phosphatase staining; scale bar, 100 µm. (D) RT-PCR detection of endogenous pluripotent mRNAs *POU5F1*, *SOX2*, *NANOG*, *KLF4*, *LIN28A*, and *MYC*. *ACTB* was used as a reference gene. (E) Amplification of EBNA and oriP in RhiPSCs, no template control (NTC), and the rhesus embryonic stem cell line r420 (RhESC). Plasmid EM2K was used as a positive control (PC) for the detection of EBNA and oriP. (F) Positive immunofluorescence staining for pluripotency proteins SOX2 (green), OCT4 (red), and NANOG (far red), colocalized with DAPI (blue). Scale bar, 100 µm. (G) Flow cytometry-detected APC-A+ cells were 93.6% positive for SOX2, 89.3% positive for OCT4, and 95.8% positive for NANOG. (H) Short tandem repeat (STR) analysis confirmed (+) RhiPSC line identity. Mycoplasma (Myco.), Herpes B Virus (HBV), Simian Immunodeficiency Virus (SIV), Simian T-Lymphotropic Virus (STLV), and Simian Retrovirus (SRV) were not detected (-). (I) Normal 42,XX karyotype. (J) H&E staining of a teratoma containing endoderm, mesoderm, and ectoderm. Scale bar, 100 µm.

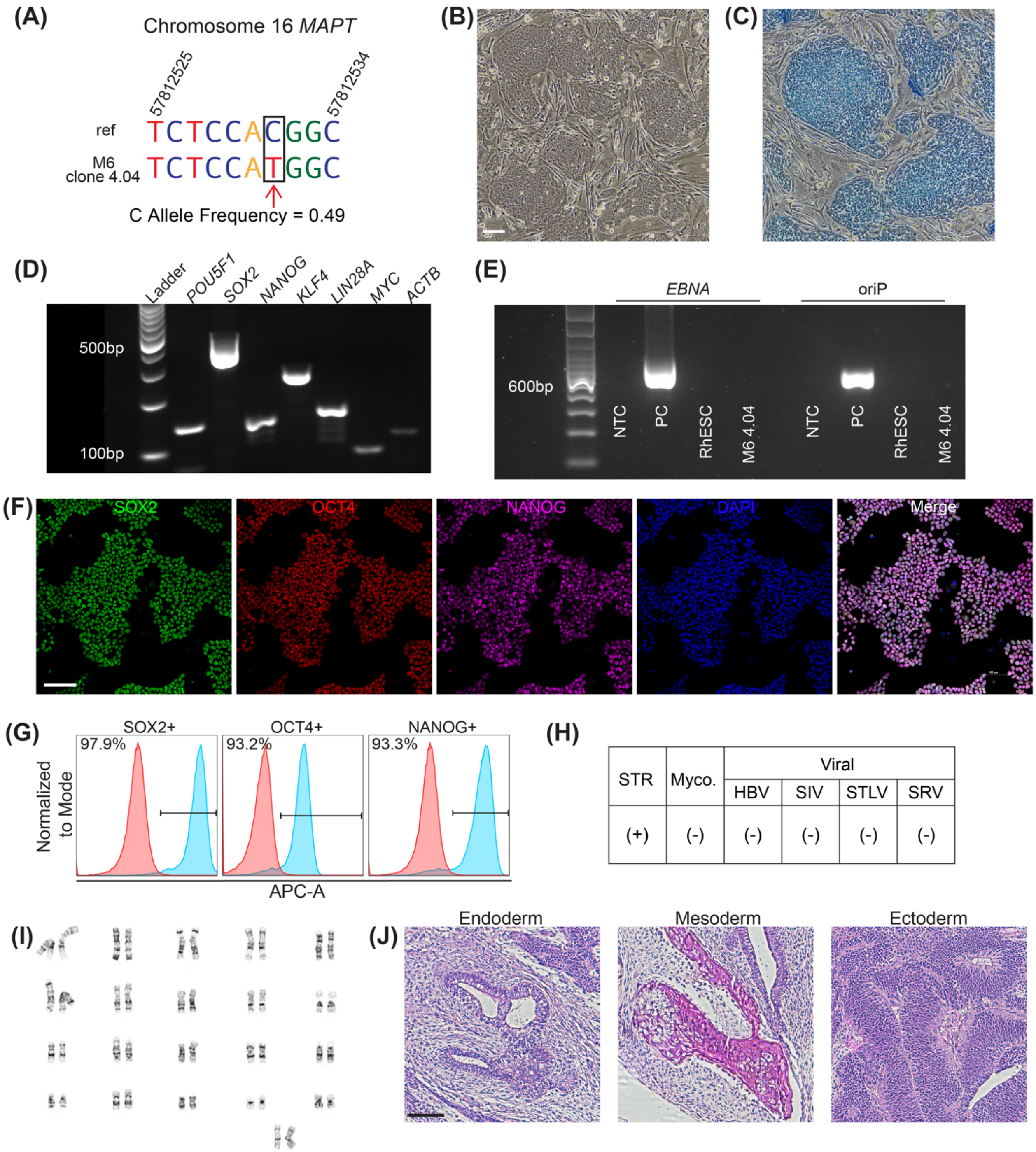

Supplementary Figure 8. Characterization of RhiPSC line M6 clone 4.04. (A) Amplicon sequencing of *MAPT* R406W with mutant T allele indicated in the black box with a red arrow compared to reference genome (ref). (B) Phase contrast image of colonies grown on MEFs and (C) positive alkaline phosphatase staining; scale bar, 100 µm. (D) RT-PCR detection of endogenous pluripotent mRNAs *POU5F1*, *SOX2*, *NANOG*, *KLF4*, *LIN28A*, and *MYC*. *ACTB* was used as a reference gene. (E) Amplification of EBNA and oriP in RhiPSCs, no template control (NTC), and the rhesus embryonic stem cell line r420 (RhESC). Plasmid EM2K was used as a positive control (PC) for the detection of EBNA and oriP. (F) Positive immunofluorescence staining for pluripotency proteins SOX2 (green), OCT4 (red), and NANOG (far red), colocalized with DAPI (blue). Scale bar, 100 µm. (G) Flow cytometry-detected APC-A+ cells were 97.9% positive for SOX2, 93.2% positive for OCT4, and 93.3% positive for NANOG. (H) Short tandem repeat (STR) analysis confirmed (+) RhiPSC line identity. Mycoplasma (Myco.), Herpes B Virus (HBV), Simian Immunodeficiency Virus (SIV), Simian T-Lymphotropic Virus (STLV), and Simian Retrovirus (SRV) were not detected (-). (I) Normal 42,XX karyotype. (J) H&E staining of a teratoma containing endoderm, mesoderm, and ectoderm. Scale bar, 100 µm.

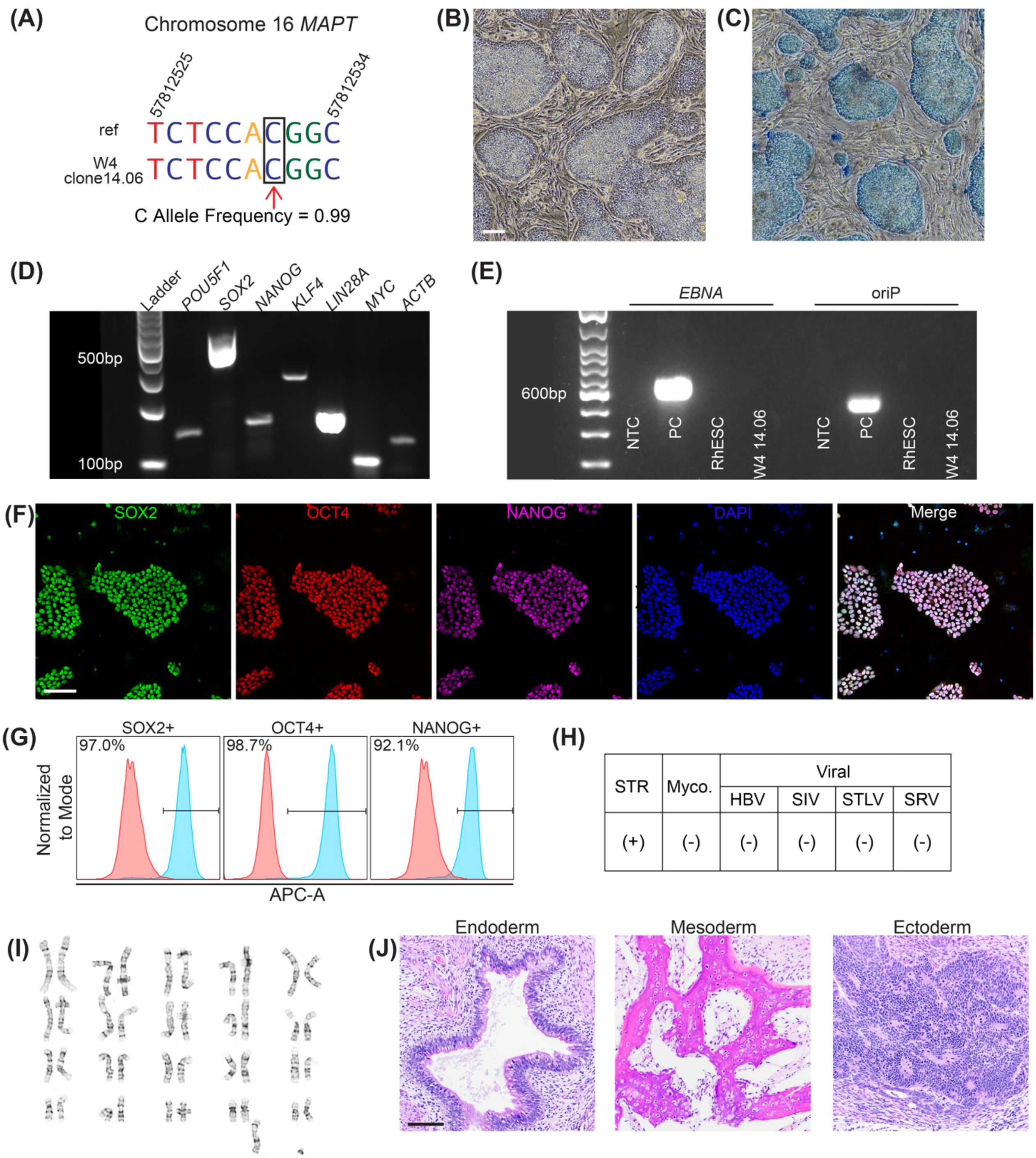

Supplementary Figure 9. Characterization of RhiPSC line W4 clone 14.06. (A) Amplicon sequencing of *MAPT* R406W with wild type C allele indicated in the black box with a red arrow compared to reference genome (ref). (B) Phase contrast image of colonies grown on MEFs and (C) positive alkaline phosphatase staining; scale bar, 100 µm. (D) RT-PCR detection of endogenous pluripotent mRNAs *POU5F1*, *SOX2*, *NANOG*, *KLF4*, *LIN28A*, and *MYC*. *ACTB* was used as a reference gene. (E) Amplification of EBNA and oriP in RhiPSCs, no template control (NTC), and the rhesus embryonic stem cell line r420 (RhESC). Plasmid EM2K was used as a positive control (PC) for the detection of EBNA and oriP. (F) Positive immunofluorescence staining for pluripotency proteins SOX2 (green), OCT4 (red), and NANOG (far red), colocalized with DAPI (blue). Scale bar, 100 µm. (G) Flow cytometry-detected APC-A+ cells were 97% positive for SOX2, 98.7% positive for OCT4, and 92.1% positive for NANOG. (H) Short tandem repeat (STR) analysis confirmed (+) RhiPSC line identity. Mycoplasma (Myco.), Herpes B Virus (HBV), Simian Immunodeficiency Virus (SIV), Simian T-Lymphotropic Virus (STLV), and Simian Retrovirus (SRV) were not detected (-). (I) Normal 42,XY karyotype. (J) H&E staining of a teratoma containing endoderm, mesoderm, and ectoderm. Scale bar, 100 µm.

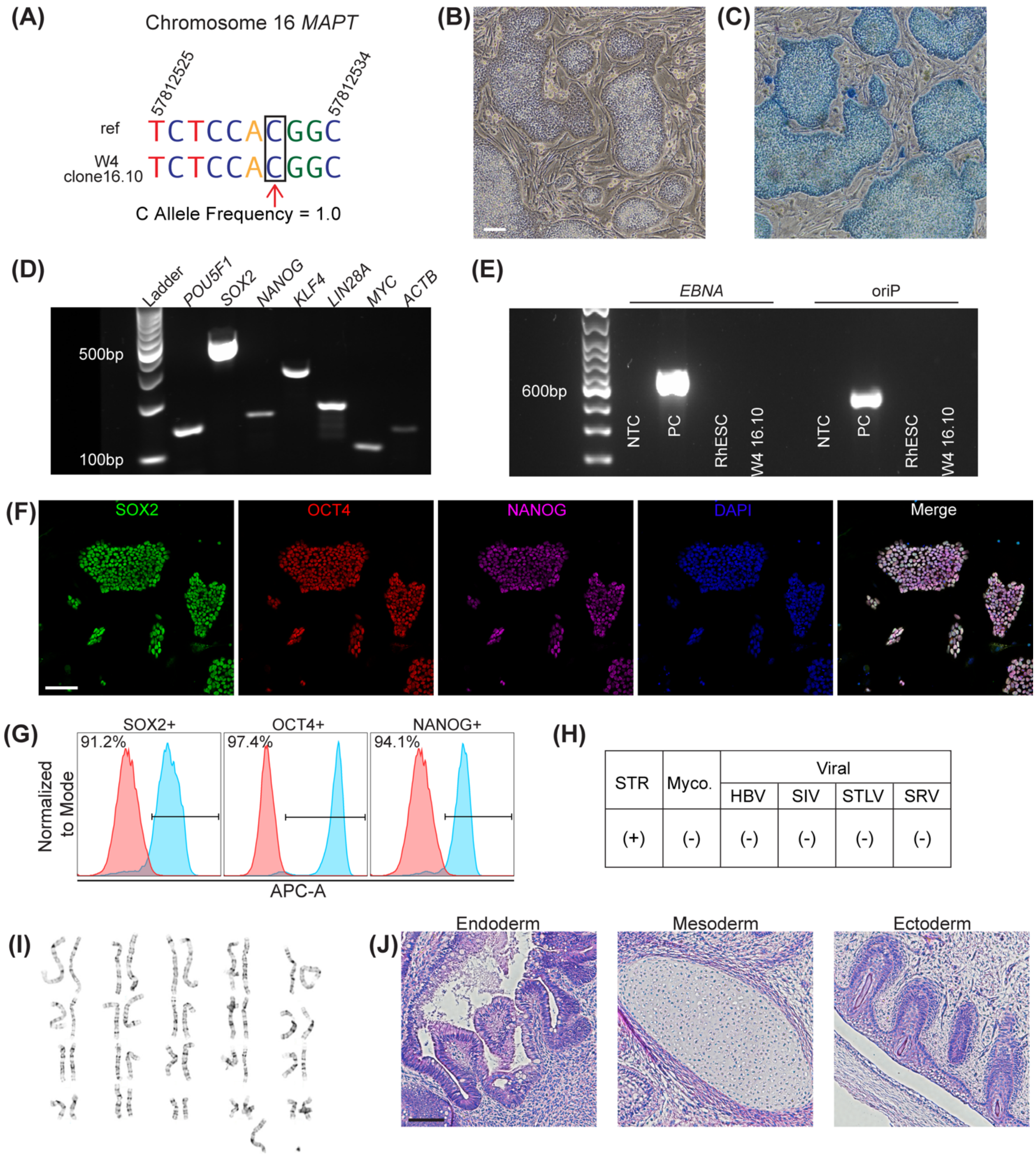

Supplementary Figure 10. Characterization of RhiPSC line W4 clone 16.10. (A) Amplicon sequencing of *MAPT* R406W with wild type C allele indicated in the black box with a red arrow compared to reference genome (ref). (B) Phase contrast image of colonies grown on MEFs and (C) positive alkaline phosphatase staining; scale bar, 100 µm. (D) RT-PCR detection of endogenous pluripotent mRNAs *POU5F1*, *SOX2*, *NANOG*, *KLF4*, *LIN28A*, and *MYC*. *ACTB* was used as a reference gene. (E) Amplification of EBNA and oriP in RhiPSCs, no template control (NTC), and the rhesus embryonic stem cell line r420 (RhESC). Plasmid EM2K was used as a positive control (PC) for the detection of EBNA and oriP. (F) Positive immunofluorescence staining for pluripotency proteins SOX2 (green), OCT4 (red), and NANOG (far red), colocalized with DAPI (blue). Scale bar, 100 µm. (G) Flow cytometry-detected APC-A+ cells were 91.2% positive for SOX2, 97.4% positive for OCT4, and 94.1% positive for NANOG. (H) Short tandem repeat (STR) analysis confirmed (+) RhiPSC line identity. Mycoplasma (Myco.), Herpes B Virus (HBV), Simian Immunodeficiency Virus (SIV), Simian T-Lymphotropic Virus (STLV), and Simian Retrovirus (SRV) were not detected (-). (I) Normal 42,XY karyotype. (J) H&E staining of a teratoma containing endoderm, mesoderm, and ectoderm. Scale bar, 100 µm.

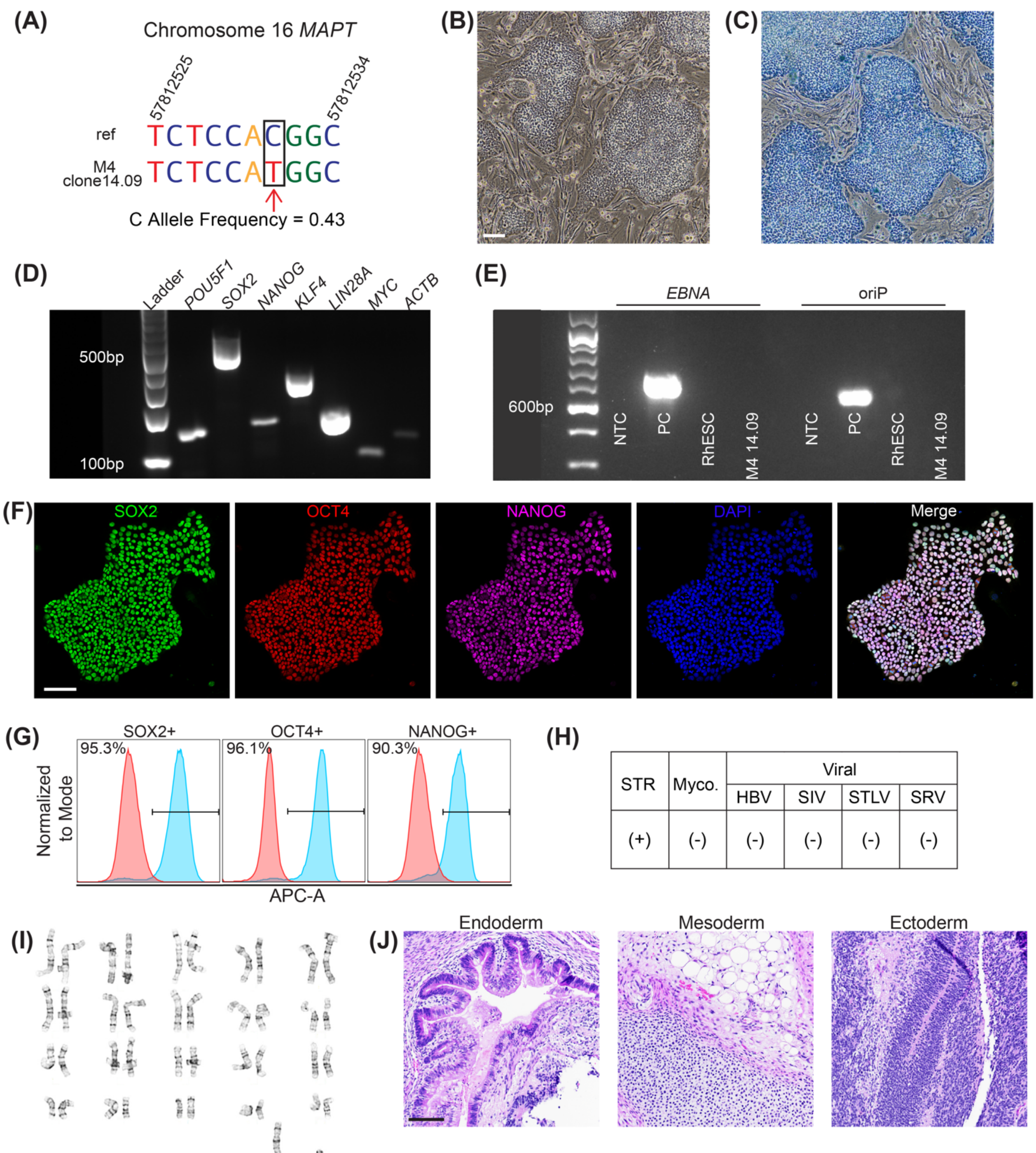

Supplementary Figure 11. Characterization of RhiPSC line M4 clone 14.09. (A) Amplicon sequencing of *MAPT* R406W with mutant T allele indicated in the black box with a red arrow compared to reference genome (ref). (B) Phase contrast image of colonies grown on MEFs and (C) positive alkaline phosphatase staining; scale bar, 100 µm. (D) RT-PCR detection of endogenous pluripotent mRNAs *POU5F1*, *SOX2*, *NANOG*, *KLF4*, *LIN28A*, and *MYC*. *ACTB* was used as a reference gene. (E) Amplification of EBNA and oriP in RhiPSCs, no template control (NTC), and the rhesus embryonic stem cell line r420 (RhESC). Plasmid EM2K was used as a positive control (PC) for the detection of EBNA and oriP. (F) Positive immunofluorescence staining for pluripotency proteins SOX2 (green), OCT4 (red), and NANOG (far red), colocalized with DAPI (blue). Scale bar, 100 µm. (G) Flow cytometry-detected APC-A+ cells were 95.3% positive for SOX2, 96.1% positive for OCT4, and 90.3% positive for NANOG. (H) Short tandem repeat (STR) analysis confirmed (+) RhiPSC line identity. Mycoplasma (Myco.), Herpes B Virus (HBV), Simian Immunodeficiency Virus (SIV), Simian T-Lymphotropic Virus (STLV), and Simian Retrovirus (SRV) were not detected (-). (I) Normal 42,XY karyotype. (J) H&E staining of a teratoma containing endoderm, mesoderm, and ectoderm. Scale bar, 100 µm.

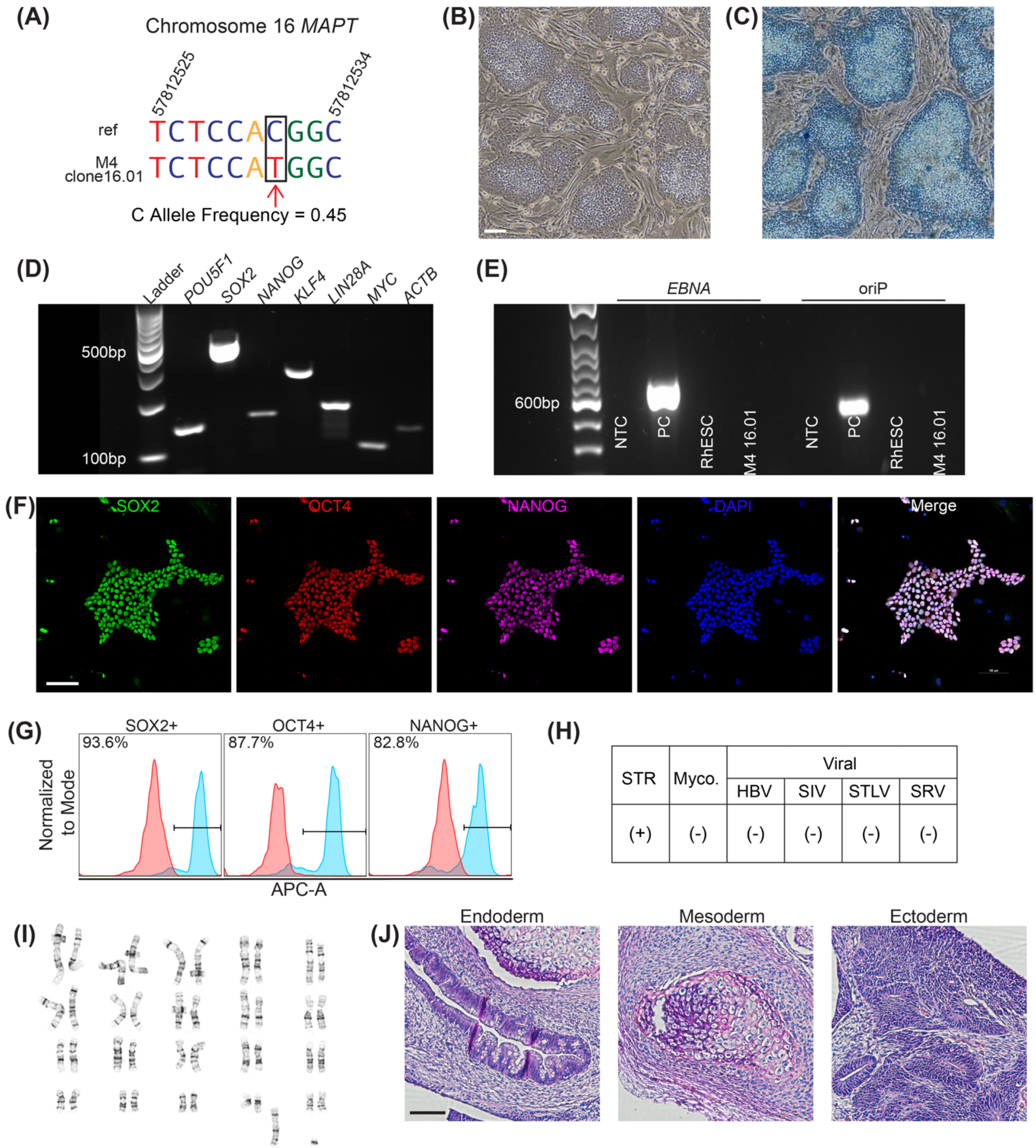

Supplementary Figure 12. Characterization of RhiPSC line M4 clone 16.01. (A) Amplicon sequencing of *MAPT* R406W with mutant T allele indicated in the black box with a red arrow compared to reference genome (ref). (B) Phase contrast image of colonies grown on MEFs and (C) positive alkaline phosphatase staining; scale bar, 100 μm. (D) RT-PCR detection of endogenous pluripotent mRNAs *POU5F1*, *SOX2*, *NANOG*, *KLF4*, *LIN28A*, and *MYC*. *ACTB* was used as a reference gene. (E) Amplification of EBNA and oriP in RhiPSCs, no template control (NTC), and the rhesus embryonic stem cell line r420 (RhESC). Plasmid EM2K was used as a positive control (PC) for the detection of EBNA and oriP. (F) Positive immunofluorescence staining for pluripotency proteins SOX2 (green), OCT4 (red), and NANOG (far red), colocalized with DAPI (blue). Scale bar, 100 μm.(G) Flow cytometry-detected APC-A+ cells were 93.6% positive for SOX2, 87.7% positive for OCT4, and 82.8% positive for NANOG. (H) Short tandem repeat (STR) analysis confirmed (+) RhiPSC line identity. Mycoplasma (Myco.), Herpes B Virus (HBV), Simian Immunodeficiency Virus (SIV), Simian T-Lymphotropic Virus (STLV), and Simian Retrovirus (SRV) were not detected (-). (I) Normal 42,XY karyotype. (J) H&E staining of a teratoma containing endoderm, mesoderm, and ectoderm. Scale bar, 100 μm.

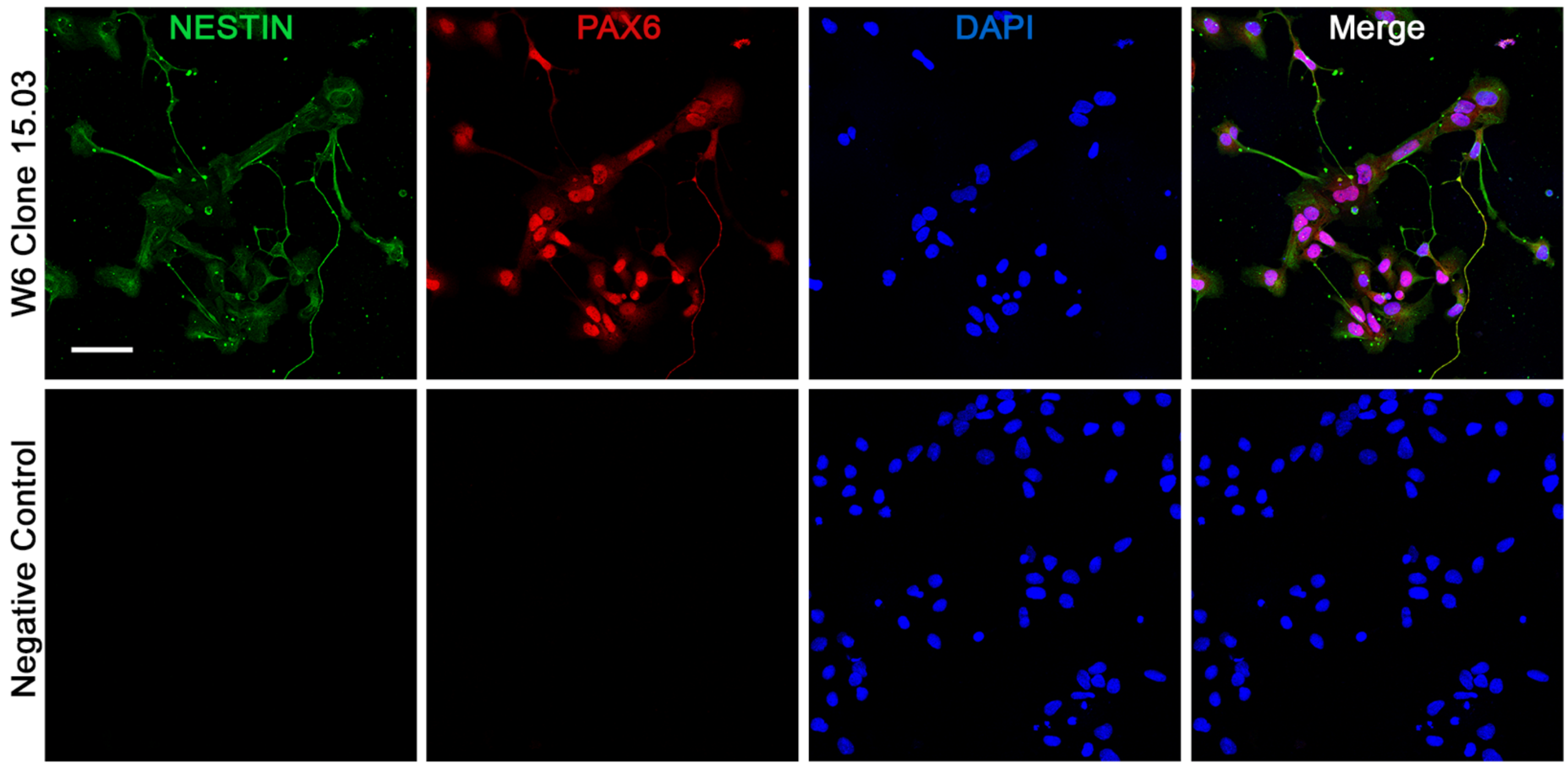

Supplementary Figure 13. RhNPCs derived from RhiPSC line W6 Clone 15.03 express NESTIN (green) and PAX6 (red) with a DAPI counterstain (blue). Primary antibody-omitted negative control immunofluorescence images from this RhNPC line are also provided. Scale bar, 100 µm.
